# ConoGPT: Fine-Tuning a Protein Language Model by Incorporating Disulfide Bond Information for Conotoxin Sequence Generation

**DOI:** 10.3390/toxins17020093

**Published:** 2025-02-17

**Authors:** Guohui Zhao, Cheng Ge, Wenzheng Han, Rilei Yu, Hao Liu

**Affiliations:** 1 College of Computer Science and Technology, Ocean University of China, Songling Road, Qingdao 266100, China; zhaoguohui@stu.ouc.edu.cn (G.Z.); hanwenzheng@stu.ouc.edu.cn (W.H.); 2 School of Medicine and Pharmacy, Ocean University of China, Songling Road, Qingdao 266100, China

**Keywords:** conotoxins, protein language model, peptide generation, activity prediction, receptors

## Abstract

Conotoxins are a class of peptide toxins secreted by marine mollusks of the Conus genus, characterized by their unique mechanism of action and significant biological activity, making them highly valuable for drug development. However, traditional methods of acquiring conotoxins, such as in vivo extraction or chemical synthesis, face challenges of high costs, long cycles, and limited exploration of sequence diversity. To address these issues, we propose the ConoGPT model, a conotoxin sequence generation model that fine-tunes the ProtGPT2 model by incorporating disulfide bond information. Experimental results demonstrate that sequences generated by ConoGPT exhibit high consistency with authentic conotoxins in physicochemical properties and show considerable potential for generating novel conotoxins. Furthermore, compared to models without disulfide bond information, ConoGPT outperforms in terms of generating sequences with ordered structures. The majority of the filtered sequences were shown to possess significant binding affinities to nicotinic acetylcholine receptor (nAChR) targets based on molecular docking. Molecular dynamics simulations of the selected sequences further confirmed the dynamic stability of the generated sequences in complex with their respective targets. This study not only provides a new technological approach for conotoxin design but also offers a novel strategy for generating functional peptides.

## 1. Introduction

Conotoxins are a class of peptide toxins secreted by marine mollusks of the genus Conus. These peptides are rich in disulfide bonds and exhibit highly specific mechanisms of action, targeting acetylcholine receptors, subtypes of other neurotransmitter receptors, as well as sodium, potassium, and calcium ion channels. Their high specificity and unique mechanisms make conotoxins attractive candidates for therapeutic applications [1,2]. For instance, the drug Ziconotide, derived from conotoxins, has been approved for treating severe chronic pain [3]. Due to their remarkable bioactivity and potential pharmaceutical value, conotoxins are a major focus in protein engineering and drug discovery. However, traditional methods of obtaining conotoxins, such as extraction from natural sources [4,5] or chemical synthesis [6], are costly, time-consuming, and limit the exploration of the vast sequence diversity of conotoxins. Consequently, there is an urgent need for advanced deep learning approaches to efficiently discover and generate bioactive conotoxins.

In recent years, the development of generative artificial intelligence has revolutionized the design of drugs [7], proteins [8], and peptides [9,10,11]. In the field of peptide generation, the most widely used frameworks include generative adversarial networks (GANs) [12,13,14], variational autoencoders (VAEs) [15,16,17], and diffusion models [18].

GANs, particularly bidirectional conditional GANs (BiCGANs) [13], have shown promise in addressing the unique challenges of designing antimicrobial peptides by mapping peptide sequences into the latent space of the generator. However, GANs are prone to mode collapse, leading to reduced diversity in generated molecules, and their training process is often unstable and difficult to optimize. VAEs, especially their conditional variants (cVAEs), are also at the forefront of peptide generation research. While VAEs probabilistically map input peptide data into a latent space, cVAEs enhance specificity and controllability in peptide generation by incorporating conditional information. Studies have demonstrated the ability of modified cVAEs to generate peptides with desirable properties, particularly in the context of antimicrobial peptide design [19]. However, the reliability of cVAE-generated sequences remains a challenge. Recent studies utilizing diffusion models have demonstrated their potential to generate antimicrobial peptides [18]. Although diffusion models excel in generating complex sequences, their high computational cost, slower generation speed, and sensitivity to model parameters limit their broader application.

In contrast, protein language models trained on large-scale protein databases capture deep patterns and structural information within protein sequences, enabling the generation of diverse, high-quality sequences. Recent advances have explored the use of large language models for protein design. ProGEN [20], for example, employs a 1.2B parameter language model to controllably generate proteins with specified molecular functions or cellular components. ProtGPT2 [21], a GPT2-based language model, has been trained on protein sequence space to generate novel proteins adhering to natural protein principles. Furthermore, fine-tuning pre-trained language models on specific datasets has been successfully applied in tasks such as enzyme design [22] and post-translational modification prediction [23], making them ideal for conotoxin generation.

Mature conotoxins typically contain multiple disulfide bonds, which are critical for their structural stability and functional activity [24]. Disulfide bonds not only maintain the three-dimensional structure of conotoxins but also directly influence their binding specificity and bioactivity toward target receptors. Therefore, incorporating disulfide bond information in conotoxin generation tasks is essential for capturing their key structural characteristics.

To address these challenges, we propose ConoGPT, a conotoxin generation model that fine-tunes ProtGPT2 with disulfide bond information to generate bioactive and structurally ordered conotoxin sequences. By introducing disulfide bond annotations in the training data, our approach aims to investigate the potential influence of disulfide information on sequence generation. This strategy enables the model to better capture the critical structural features of conotoxins. Experimental results demonstrate that the generated sequences exhibit high consistency in physicochemical properties compared to natural conotoxins and perform well in structural prediction, activity evaluation, and molecular docking experiments. This study provides an efficient and reliable method for the generation and optimization of conotoxin sequences, advancing their application in drug development and offering new directions for generating other functional protein sequences.

## 2. Results

### 2.1. Comparison of Generated Sequences with Real Conotoxin Sequences

To assess the biological plausibility and effectiveness of the sequences generated by the ConoGPT model, 10,000 sequences were randomly generated and compared across multiple dimensions with known real conotoxin sequences, primarily sourced from the ConoServer database [25]. The analyses encompassed amino acid composition, physicochemical properties, Shannon Entropy of subsequence distributions, cysteine framework distributions, and sequence similarity scores.

#### 2.1.1. Amino Acid Composition and Physicochemical Properties

**Figure 1:**
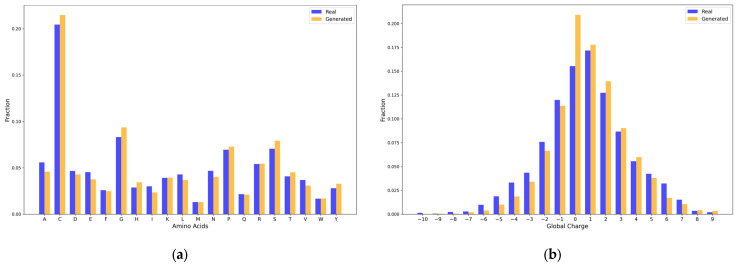

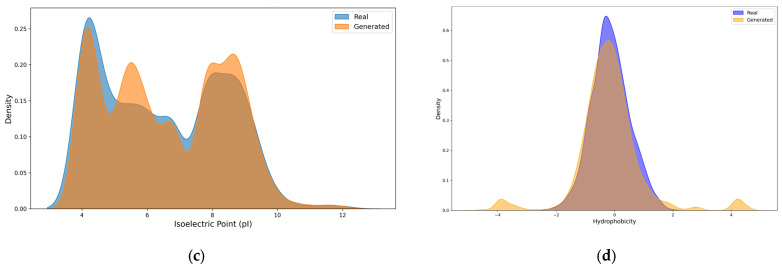
Comparison of amino acid composition and physicochemical properties between sequences generated by the ConoGPT model and real conotoxins. (**a**) Amino acid composition shows a high degree of similarity, particularly in the proportions of cysteine, glutamic acid, and aspartic acid. (**b**) Charge distributions of generated and real sequences are highly overlapping, with most sequences having a charge between 0 and +3. (**c**) Isoelectric point (pI) analysis indicates similar distributions across the range of 4–10, with peaks around 6–8. (**d**) Hydrophobicity profiles of generated and real sequences are centered near zero, indicating comparable properties critical for conotoxin stability and function.

The amino acid composition analysis (Figure 1a) revealed that the generated sequences closely resemble the real conotoxins, especially in the proportions of key amino acids such as cysteine(C) and glutamic acid(E), which play critical roles in conotoxin structure and function [26]. For charge distribution (Figure 1b), both the generated and real sequences exhibited similar profiles, with the majority of sequences having a charge between 0 and +3. This overlap indicates that the generated sequences possess charge properties comparable to real conotoxins. However, the generated sequences show a slightly higher proportion at zero charge, which may be attributable to a bias toward neutral charges in the model itself. Isoelectric point (pI) analysis (Figure 1c) showed that both groups had overlapping distributions in the range of 4 to 10, with a peak at 6–8, further confirming the reasonable physicochemical properties of the generated sequences. Lastly, we computed the hydrophobicity scores of both the generated and real conotoxins using the Kyte–Doolittle hydropathy index. The hydrophobicity distributions (Figure 1d) demonstrated a high degree of similarity, with both groups centered near zero, a property critical for the stability and functionality of conotoxins. Collectively, these results underscore the ability of the ConoGPT model to capture the essential features of real conotoxins in its generated sequences.

#### 2.1.2. Shannon Entropy of Subsequence Distribution

**Figure 2:**
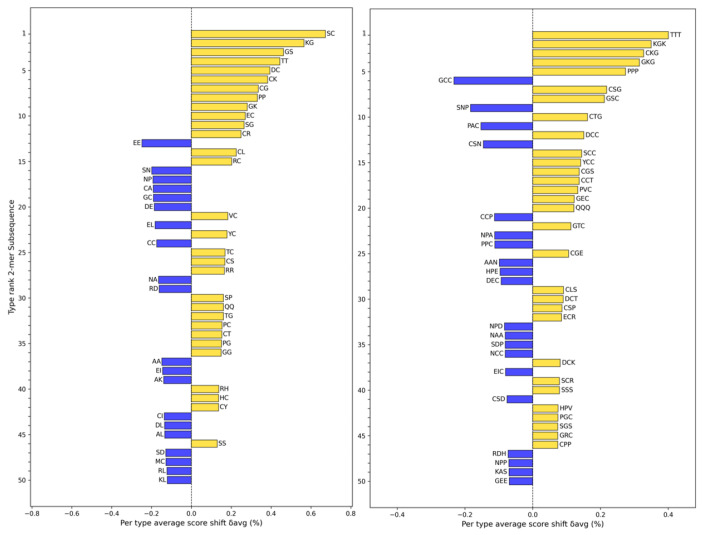
Shannon’s entropy divergence between the distributions of length 2 (**left**) and length 3 (**right**) subsequences of FASTA characters in conotoxins from the training set (real) or conotoxins created by the generator (generated). Blue bars indicate a greater prevalence of a particular subsequence in real conotoxins, while gold bars indicate a greater prevalence in generated conotoxins. For length 2 subsequences, the average entropy was 7.94 for real sequences and 7.79 for generated sequences, while for length 3 subsequences, the average entropy was 11.42 for real sequences and 11.38 for generated sequences. For reference, the distribution of subsequences drawn from uniformly random sequences results in a maximum entropy of ~8.64 for length 2 subsequences and ∼12.97 for length 3 subsequences. Both groups in both plots feature a lower entropy than the maximum; thus, we should expect to see meaningful structures in each group.

Shannon Entropy [27] is a measure from information theory used to quantify the diversity or uncertainty of a distribution. We evaluate the subsequence distribution of generated and real sequences by calculating their Shannon Entropy. In Figure 2, we provide word shifts between generated and real conotoxin sequences for subsequences of lengths 2 and 3. The subsequences more common in the generated peptides predominantly involve one or more instances of cysteine (C) or glycine (G). Conversely, subsequences enriched in real peptides are more likely to involve glutamic acid (E) or alanine (A). These compositional differences reflect unique sequence preferences between the two groups.

The preference for C and G in the generated sequences aligns with structural features such as disulfide bond formation (C) and increased flexibility (G). Meanwhile, the enrichment of E and A in real sequences suggests contributions to negative charge properties (E) and the formation of hydrophobic cores (A).

#### 2.1.3. Cysteine Framework Distribution Analysis

**Figure 3:**
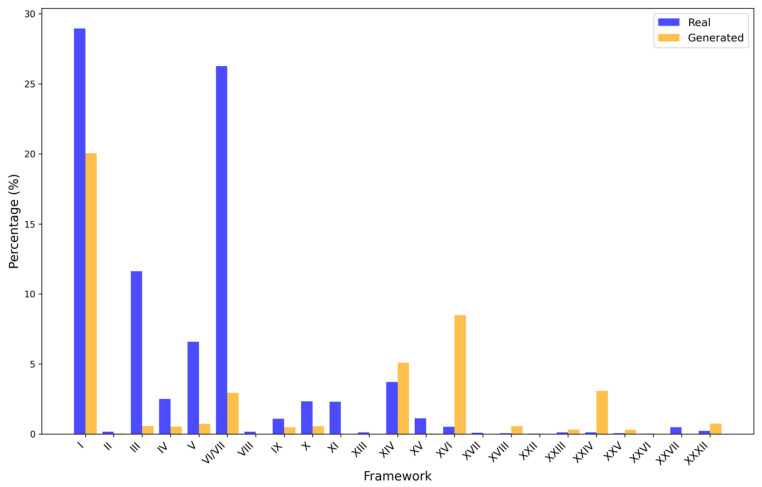
Cysteine framework distributions in generated and real conotoxin sequences. The cysteine connectivity is not known for all frameworks; only frameworks I, IV, V, VI/VII, IX, X, XI, and XIV have confirmed connectivity.

Cysteine frameworks are critical features of conotoxins, as they determine the spatial conformation and functional stability of the peptides. As shown in Figure 3, there are notable differences in the framework distributions between the generated and real sequences. In real conotoxin sequences, the predominant frameworks are type I (CC-C-C) and VI/VII (C-C-CC-C-C), accounting for 29.5% and 26.3% of the distribution, respectively. In contrast, the proportion of type I frameworks in the generated sequences is 21.1%, while the VI/VII frameworks show a significant reduction, accounting for only 3.2%. Additionally, the generated sequences exhibit a higher proportion of type XVI (C-C-CC) frameworks (9.1%) and type XIV (C-C-C-C) frameworks (5.5%), which are relatively less frequent in the real sequences. This may suggest that the model prefers to generate sequences containing two disulfide bonds.

Notably, 52.7% of the generated sequences may contain previously undiscovered types of cysteine frameworks, suggesting the potential for developing novel conotoxins with a new structure.

#### 2.1.4. Diversity Analysis

**Figure 4:**
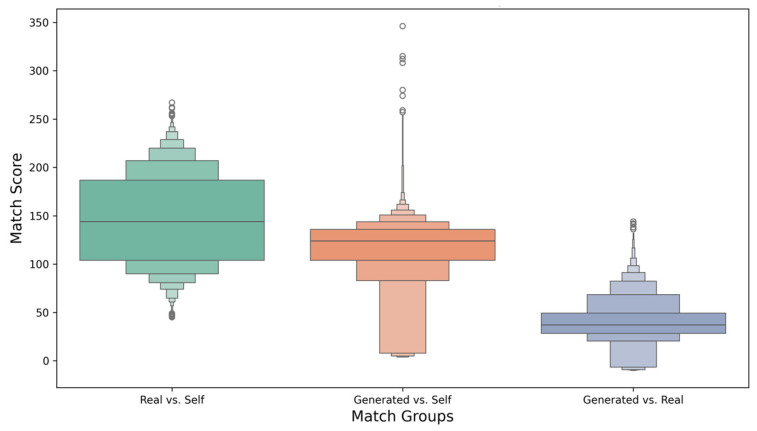
Distribution of sequence match scores across three groups: “Real vs. Self”, “Generated vs. Self”, and “Generated vs. Real”. The “Real vs. Self” group displays a higher median score with a concentrated distribution, while the “Generated vs. Self” group shows greater variability and diversity. The “Generated vs. Real” group demonstrates the lowest median score, emphasizing the novelty of generated sequences compared to real conotoxins.

When proposing candidate conotoxins, it is important that the generated candidates are diverse as a population and novel relative to known conotoxins. As shown in Figure 4, we analyzed the distribution of sequence match scores across three groups: “Real vs. Self”, “Generated vs. Self”, and “Generated vs. Real”. Match scores were calculated using global sequence alignment based on the BLOSUM62 [28] substitution matrix. The “Real vs. Self” group exhibited a high median match score (~150) with a relatively narrow distribution, indicating strong internal consistency among real conotoxin sequences. The symmetric distribution and a small number of outliers further suggest limited sequence variability within the real conotoxin dataset. In contrast, the “Generated vs. Self” group displayed a broader and more dispersed distribution with a lower median match score (~100), reflecting greater diversity among the generated sequences compared to the real conotoxins. Additionally, some outliers with high match scores indicate that certain generated sequences are highly similar to others within the generated dataset. The “Generated vs. Real” group exhibited the lowest median match score (~50) and a narrower distribution skewed toward lower match scores. This highlights the substantial differences between generated and real conotoxin sequences, underscoring the novelty and uniqueness of the generated sequences.

#### 2.1.5. Activity Assessment

**Figure 5:**
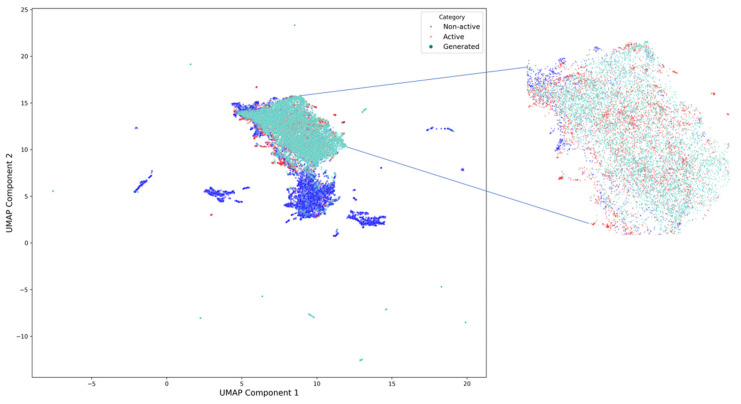
UMAP visualization of the embedding space for generated sequences (mint green) and real sequences, with active real sequences shown in red and inactive real sequences shown in blue. The magnified view highlights the overlap between generated sequences and active real sequences, demonstrating the ability of the model to generate sequences closely resembling real active ones while also exploring new regions of the embedding space.

In order to evaluate the potential biological functionality of the generated sequences, we employed a publicly available peptide activity prediction framework, UniDL4BioPep [29], which was retrained specifically for a binary classification task tailored to conotoxins. The model predicted that 81.86% of the generated sequences are likely to be bioactive, indicating that the fine-tuned model incorporating disulfide bond information performs well in terms of generating sequences with potential activity. To provide a more intuitive representation of the feature distribution of generated and real sequences in the embedding space, we used UMAP [30] to visualize the high-dimensional embeddings in reduced dimensions, as shown in Figure 5. In the UMAP plot, the majority of generated sequences (mint green) cluster within the regions occupied by real active sequences (red), demonstrating high similarity between the generated sequences and real active sequences in the embedding space. Meanwhile, some generated sequences extend into new regions of the embedding space, suggesting that the model possesses innovation capabilities and can explore potential sequence features not present in the training dataset.

### 2.2. Comparative Analysis of Model Performance

To further investigate the impact of incorporating disulfide bond information on the performance of the generation model, we compared the properties of sequences generated by the baseline model (without disulfide bond information) and the improved model (with disulfide bond information). The analysis focused on four key aspects: perplexity, structural order (measured by pLDDT values), cysteine framework distribution, and activity prediction outcomes. These comparisons comprehensively evaluate the influence of disulfide bond information on the characteristics of the generated sequences.

#### 2.2.1. Perplexity Comparison

**Figure 6:**
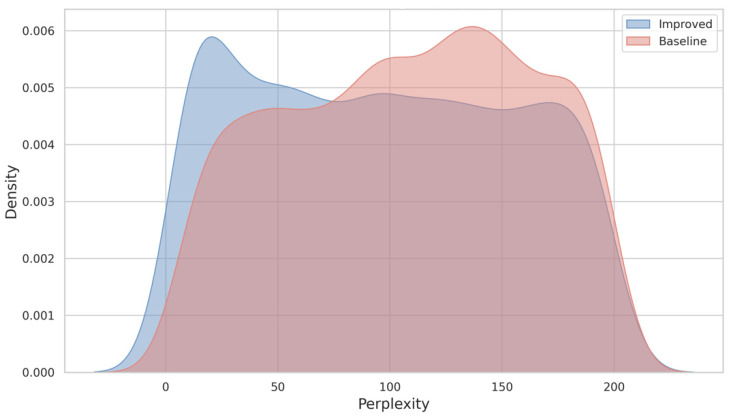
Density comparison of perplexity distributions for sequences generated by the baseline model (red) and the improved model (blue). The improved model demonstrates slightly lower average perplexity compared to the baseline model.

Perplexity is a crucial metric for evaluating the predictive capability of language models, reflecting the model’s confidence in sequence generation. Lower perplexity generally indicates that the generated sequences better align with the distribution of the training data. Figure 6 compares the perplexity distributions of sequences generated by the baseline model (without disulfide bond information) and the improved model (with disulfide bond information). The improved model demonstrates a slightly lower average perplexity (79.79) compared to the baseline model (81.83). This marginal improvement suggests that the inclusion of disulfide bond information helps the model better capture the structural and sequential characteristics of conotoxins. The perplexity density curves of both models, however, exhibit significant overlap, indicating similar predictive capabilities. Notably, the perplexity distributions for both models are concentrated within the range of 50 to 150, with the improved model showing a slightly higher density around lower perplexity values.

#### 2.2.2. Evaluation of Structural Orderliness in Generated Sequences

An essential characteristic to consider when designing conotoxin sequences is their ability to fold into stable and ordered structures. We employed ESMFold [31] to predict the three-dimensional structures of the generated sequences and calculated the pLDDT score for each sequence. A pLDDT score between 70 and 90 indicates a well-modeled, ordered structure, while lower scores (pLDDT < 50) are typically associated with disordered regions [32].

To ensure the quality of the generated sequences and the validity of subsequent analyses, the top 1000 sequences with the lowest perplexity were selected from the 10,000 sequences generated by each model. By averaging the results of five independent generation trials, we minimized experimental randomness and ensured robust conclusions. Using box plots, we visually compared the pLDDT distribution characteristics of the two models, and the proportion of high-confidence sequences (pLDDT > 0.7) was also calculated.

**Figure 7:**
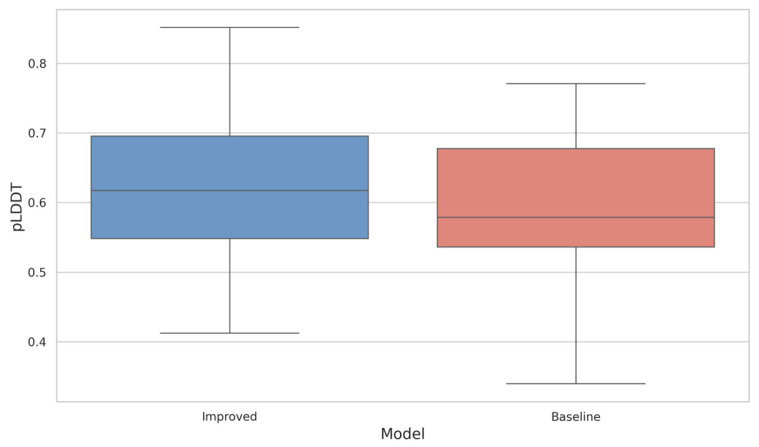
Box plot comparison of pLDDT distributions for sequences generated by the baseline model and the improved model.

Below, Figure 7 presents the box plot comparison of pLDDT distributions, and Table 1 summarizes the proportion of high-confidence sequences for both models.

#### 2.2.3. Evaluation of Cysteine Framework Distributions

**Figure 8:**
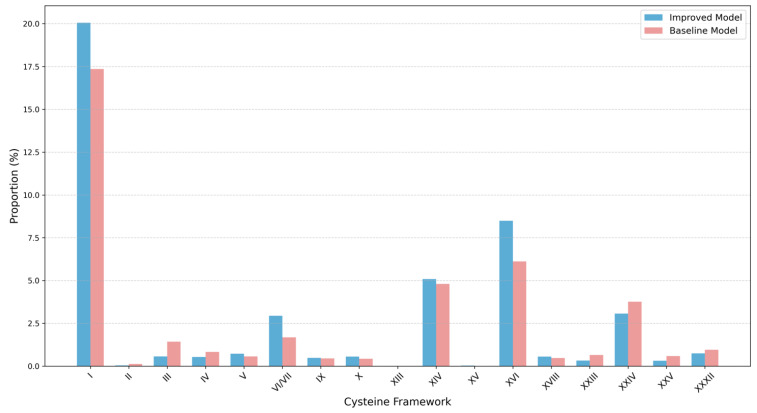
Cysteine framework distribution generated by the improved model (blue) and the baseline model (pink). The improved model demonstrates a higher proportion in the vast majority of known cysteine-connected frameworks (I, V, VI/VII, IX, X, XI, XIV, as mentioned in Section 4.1).

The cysteine framework of conotoxins is closely related to their disulfide connectivity, which forms the basis for the improved model’s integration of disulfide bond information. To assess the influence of this improvement, we compared the distribution of known cysteine frameworks between the improved model and the baseline model, with the results shown in Figure 8.

#### 2.2.4. Analysis of Activity Prediction

To further evaluate the potential bioactivity of the generated sequences, we employed a retrained UniDL4BioPep activity prediction model to assess sequences generated by the two models. UniDL4BioPep, a framework specifically designed for peptide activity prediction, was retrained in this study for a binary classification task tailored to conotoxins.

The results revealed that 81.86% of the sequences generated by the improved model were predicted to be bioactive, compared to 80.26% for the baseline model. The slight difference in bioactive proportions indicates that incorporating disulfide bond information in the fine-tuning process has a minimal impact on the bioactivity potential of the generated sequences.

### 2.3. Functional Validation of Generated Sequences

Functional validation is a critical step in assessing the potential applicability of the generated conotoxin sequences. Beyond evaluating the physicochemical properties and structural stability of these sequences, it is essential to determine their ability to interact with biological targets effectively. In this study, we focused on two key validation strategies: molecular docking and molecular dynamics simulations. These methods provide insights into the binding affinities and dynamic stability of the generated sequences with relevant receptor targets, offering a comprehensive evaluation of their potential biological functions and applications.

#### 2.3.1. Analysis of Molecular Docking

To evaluate the functional potential of the sequences generated by the improved model, we conducted molecular docking analysis on 191 high-quality sequences obtained through multiple selection steps. These sequences were derived from 10,000 randomly generated sequences and underwent filtering based on perplexity (top 1000), structural stability assessment (pLDDT > 0.7), and activity prediction. The three-dimensional structures of the sequences were predicted using AlphaFold3, which ensures a reliable structural basis for subsequent docking analyses. The docking targets included common subtypes of nicotinic acetylcholine receptors (nAChRs), sodium channels (Na), calcium channels (Ca), and potassium channels (K). The docking scores obtained from LeDock [33] were used to determine the final targets. Sequences with docking scores below −8.0 kcal/mol, and with the lowest docking score among all subtypes of a given target, were assigned to that specific target.

**Figure 9:**
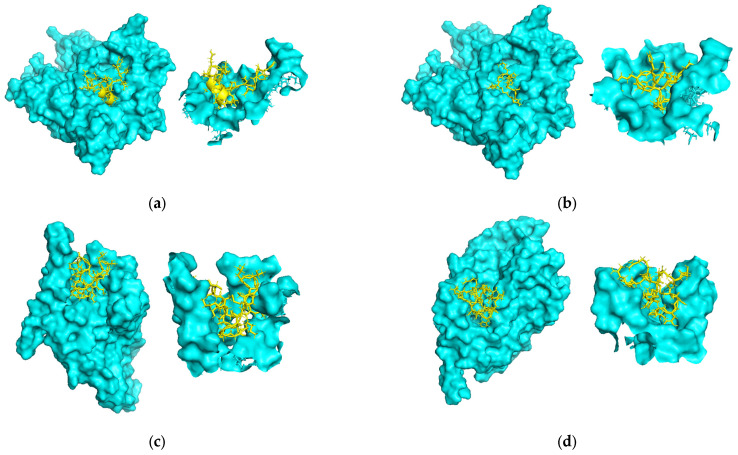
(**a**) Molecular docking result of the generated sequence YRRECCSNPACRVDHPEICG with the α9α10 subtype of nAChR. (**b**) Molecular docking result of the real conotoxin GI with the α9α10 subtype of nAChR, showing binding interactions with the same target as in (**a**). (**c**) Docking result of the generated sequence CKGKGAKCSLTSSNCCSGSCRSGKC with a calcium ion channel. (**d**) Docking result of the real conotoxin CVIA with a calcium ion channel, comparing the binding affinity and interaction sites with the same target as in (**c**).

Figure 9a shows the molecular docking result of the generated sequence YRRECCSNPACRVDHPEICG with the α9α10 subtype of nAChR, illustrating its binding interactions and conformational fit within the receptor’s binding pocket. Figure 9b presents the docking result of a real conotoxin with the α9α10 subtype of nAChR, showing that both the generated sequence and the real conotoxin bind to the same receptor pocket, further validating the accuracy of the generated sequence’s binding mode. Figure 9c presents the docking result of CKGKGAKCSLTSSNCCSGSCRSGKC with a calcium ion channel. Figure 9d shows the docking result of a real conotoxin with the same calcium ion channel, where the binding pose of the real conotoxin differs from the generated sequence, indicating that the generated sequence binds to a different region of the channel compared to the real conotoxin.

Nicotinic acetylcholine receptors (nAChRs), as classic targets of conotoxins, exhibited a significant binding preference in this study, as shown in Table 2. The majority of the generated sequences (90.57%) demonstrated strong binding affinities with nAChRs. This may be because the majority of sequences selected after filtering have cysteine connectivity patterns of C1~C3 and C2~C4, which are typical pairings for α-conotoxins and α-conotoxins target nAChRs [34].

#### 2.3.2. Analysis of Molecular Dynamics Simulation

**Figure 10:**
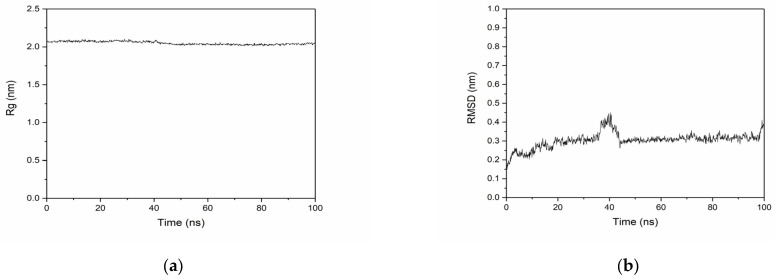

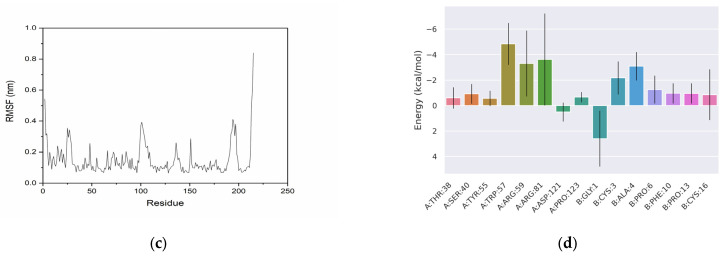
Molecular dynamics simulation of the generated sequence GCCADPKCAFNNPELC with the α3β4 subtype of nAChR. (**a**) The radius of gyration (Rg) values. (**b**) The root-mean-square deviation (RMSD) over time. (**c**) The binding free energy analysis. (**d**) The residue interaction analysis.

To further validate the functionality of the generated sequences, molecular dynamics (MDs) simulations were performed for selected sequences and their corresponding targets. Figure 10 presents the MDs simulation results for the generated sequence GCCADPKCAFNNPELC in complex with the α3β4 subtype of nAChR.

During the 100 ns simulation, the radius of gyration (Rg) values stabilized between 2.009 and 2.105 nm, with a final value of 2.057 nm at 100 ns, indicating that the protein structure remained compact. The root-mean-square deviation (RMSD) values fluctuated slightly in the first 20 ns but stabilized over time, suggesting that the system reached equilibrium.

Binding free energy analysis showed a total binding free energy of −19.45 ± 8.2 kcal/mol, confirming strong interactions between the protein and peptide. Residue interaction analysis revealed key contributions from protein residues TRP57, ARG59, and ARG81, highlighting their critical role in binding stability. Aromatic residues (such as TRP) and positively charged residues (such as ARG) often play critical roles in protein–ligand interactions [35,36,37]. We hypothesize that these residues may contribute to binding stability through different interaction mechanisms. For instance, TRP57 may stabilize the protein–peptide complex through hydrophobic interactions, while ARG59 and ARG81 may further enhance binding stability through electrostatic interactions and hydrogen bond formation.

## 3. Discussion

This study presents ConoGPT, a conotoxin generation model fine-tuned with disulfide bond information based on the ProtGPT2 framework. Through comprehensive evaluations of physicochemical properties, structural stability, and functional potential, the effectiveness of this model in generating high-quality and functional conotoxin sequences has been validated.

We have demonstrated that the sequences generated by ConoGPT exhibit high consistency with real conotoxins in terms of physicochemical properties, including amino acid composition, charge distribution, isoelectric point, and hydrophobicity. This consistency indicates that the fine-tuned model effectively captures the core features of the training data. Diversity analysis further revealed that the generated sequences display considerable diversity, highlighting the potential for discovering novel conotoxins.

From a functional perspective, the molecular docking results show a remarkable target preference of the generated sequences for nicotinic acetylcholine receptors (nAChRs). Molecular dynamics simulations, conducted on a subset of sequences, further corroborated the structural stability and target binding capabilities of these specific examples. Despite these promising results, certain limitations should be acknowledged. The current study relies on in silico evaluations without experimental validation, and the functional potential of the generated sequences remains theoretical.

## 4. Materials and Methods

### 4.1. Data Collection and Preprocessing

The conotoxin sequence data used in this study were sourced from the ConoServer and UniProt databases [38], focusing on mature peptide sequences with lengths ranging from 10 to 40 amino acids. Specifically, 3289 conotoxin sequences were extracted from the ConoServer database, while an additional 286 sequences were obtained from the UniProt database, resulting in a total dataset of 3525 conotoxin sequences.

The ConoServer database identifies 31 known cysteine frameworks, of which eight (I, IV, V, VI/VII, IX, X, XI, and XIV) have established disulfide connectivity [34]. These eight frameworks, along with their cysteine patterns, cysteine counts, disulfide connectivity, and their proportions in the dataset, are detailed in Table 3.

Disulfide pairing annotations based on these frameworks were added at the beginning of each sequence to specify cysteine connectivity, such as [C1–C4; C2–C3]. This annotation indicates that the first and fourth cysteines in the sequence form a disulfide bond, as do the second and third cysteines. These annotations provided the model with key structural information specific to conotoxin sequences. The remaining sequences, without annotations, were retained to enable the model to learn general features of conotoxins and ensure diversity in the generated sequences.

Additionally, a negative dataset was collected from the UniProt database for training the UniDL4BioPep classification model. Entries were limited to peptides with a maximum length of 40 amino acids. Any entries containing the keywords “toxic” or “conus” were excluded to avoid overlap with conotoxin-related sequences.

### 4.2. Generation Model

This study employs ProtGPT2 as the foundational model for generating conotoxin sequences. ProtGPT2, a GPT-2-based autoregressive language model, demonstrates robust capabilities in protein sequence generation. The model architecture is based on the GPT2-large Transformer, comprising 36 layers, a model dimensionality of 1280, and 20 multi-head attention units, each with a head dimension of 64, resulting in a total of 738 million parameters. To explore the potential impact of disulfide bond annotations on the generated sequences, this study incorporates such annotations as prefixes in the training data. Although these annotations exist only as prefixes to the input sequences, they provide prior guidance through the model’s attention mechanism and contextual learning capabilities. This enables the model to learn implicit relationships between disulfide bonds and sequence features, aiding in the generation of more structurally ordered conotoxin sequences.

The training objective minimizes the cross-entropy loss, defined as follows:

(1) $$L=-\sum_{t=1}^{T}\;\log\;p\left(y_{t}|y_{<t}\right)$$

where $y_{t}$ represents the *t*−th token in the generated sequence, and $y_{<t}$ denotes the context sequence comprising the preceding *t*−1 tokens. Disulfide bond annotations, included as part of the input sequence, are learned alongside subsequent tokens. Through contextual dependencies, the model establishes implicit associations between the annotations and the sequence. The fine-tuning process was conducted on two NVIDIA A6000 GPUs.

After fine-tuning the model, random sampling is employed to generate candidate conotoxin sequences. To balance the diversity and quality of the generated sequences, several key parameters are configured. The repetition penalty is set to 1.2 to mitigate token repetition during generation. The Top-k parameter is set to 950, restricting sampling at each step to the top 950 tokens with the highest probabilities. These parameter settings have been shown to achieve an optimal balance between diversity and quality in the generated sequences.

### 4.3. pLDDT Prediction

To evaluate the potential structural properties of the generated conotoxin sequences, we employed ESMFold, a highly efficient protein structure prediction tool that leverages protein language models to rapidly generate three-dimensional structures for target sequences. The pLDDT score serves as a proxy for structural orderliness, representing the Predicted Local Distance Difference Test. It corresponds to the model’s predicted score on the IDDT-Cα metric. A pLDDT score between 70 and 90 indicates a well-modeled, ordered structure, while lower scores (pLDDT < 50) are typically associated with disordered regions.

### 4.4. Activity Prediction

To evaluate the potential biological activity of the generated sequences, we employed the UniDL4BioPep model based on ESM-2 embedding features for activity prediction. UniDL4BioPep is a binary classification model specifically designed to effectively distinguish between bioactive and non-bioactive sequences. For this study, the model was retrained to suit the conotoxin activity prediction task. The training data comprised active sequences extracted from real conotoxin sequences and artificially generated inactive sequences. The dataset was split into training and testing sets at an 8:2 ratio to ensure both model effectiveness and generalization capability.

The model utilized a convolutional neural network (CNN) architecture as the core of the classification model. It consisted of two one-dimensional convolutional layers, pooling layers, fully connected layers, and a softmax output layer. The convolutional layers captured local patterns within the embedding features, the pooling layers reduced dimensionality to decrease computational complexity, and the fully connected layers integrated global features for final classification. The model’s performance was assessed through 10-fold cross-validation, with metrics including accuracy (ACC), balanced accuracy (BACC), sensitivity (Sn), specificity (Sp), Matthews correlation coefficient (MCC), and area under the curve (AUC). The results are presented in Table 4.

### 4.5. Molecular Docking

To validate the potential functionality of the generated sequences, molecular docking experiments were conducted using LeDock software (Linux_x86_64 version). LeDock is a highly efficient molecular docking tool capable of rapidly predicting binding modes and binding free energies between molecules and their targets. To ensure accurate input structures for docking, the three-dimensional structures of the filtered generated sequences and the structures of all docking targets were predicted using AlphaFold3. AlphaFold3 [39], a deep learning-based protein structure prediction tool, produces high-confidence three-dimensional conformations of sequences.

The docking targets included five subtypes of nicotinic acetylcholine receptors (nAChRs), specifically α3β2, α3β4, α4β2, α7 and α9α10, along with several specific ion channels. These ion channels comprised calcium ion channels (Ca), including L-type (Ca_v_1.1), P/Q-type (Ca_v_2.1), and N-type (Ca_v_2.2); sodium ion channels, including Na_v_1.2, Na_v_1.3, Na_v_1.4, and Na_v_1.7; and voltage-gated potassium channels (K_v_), such as K_v_1.3 and K_v_2.1. In the experimental design, the selected generated sequences were first subjected to AlphaFold for three-dimensional structure prediction. The resulting structures were optimized and then input into LeDock for docking analysis with the target receptors. Each sequence was docked against multiple receptor subtypes, with binding free energies (measured in kcal/mol) and other related parameters recorded.

### 4.6. Molecular Dynamics Simulation

In this study, molecular dynamics (MDs) simulations were employed to analyze the dynamic behavior of certain peptide–target complexes obtained from molecular docking. MDs simulations provide insights into the dynamic behavior of molecules in their natural environment, enabling the evaluation of the stability of peptide–target complexes.

The peptide–target complexes derived from the docking results were selected for MDs simulations using Gromacs software (2022.2 version). The Amber99sb-ildn force field was applied to proteins and peptides, and the TIP3P water model was used to solvate the system. A cubic simulation box of size 10 × 10 × 10 nm^3^ was constructed, with at least 1.2 nm between the protein and the box edges. The system was neutralized with counterions to maintain overall charge neutrality. Electrostatic interactions were calculated using the Particle Mesh Ewald (PME) method, and energy minimization was performed using the steepest descent algorithm for up to 50,000 steps. The cutoff distances for Coulomb and van der Waals interactions were set to 1 nm.

After energy minimization, the system was equilibrated under constant volume and temperature (NVT) conditions, followed by constant pressure and temperature (NPT) conditions. The final production run consisted of a 100 ns MDs simulation at 300 K and 1 bar. A cutoff distance of 10 Å was used for non-bonded interactions. The Langevin thermostat was applied to maintain a stable temperature, while the Berendsen barostat was used to regulate pressure.

## Figures and Tables

**Table 1:** Proportion of high-confidence sequences (pLDDT > 0.7) generated by the baseline and improved models.

Model	Percentage with pLDDT > 0.7 (%)	Mean pLDDT
Improve	21.08 ± 0.56	0.6090 ± 0.0033
Baseline	14.52 ± 1.23	0.5820 ± 0.0019

**Table 2:** Distribution of docking results across receptor targets for the 191 generated sequences.

Target	Number of Sequences	Proportion (%)
nAChR	173	90.57
Na	1	0.52
Ca	2	1.05
K	0	0.00
Unknown	15	7.85

**Table 3:** Distribution of cysteine frameworks with known disulfide bond connectivity.

Framework	Cysteine Pattern	Cysteines	Connectivity	Percentage (%)
I	CC-C-C	4	I–III, II–IV	28.95
IV	CC-C-C-C-C	6	I–V, II–III, IV–VI	2.50
V	CC-CC	4	I–III, II–IV	6.59
VI/VII	C-C-CC-C-C	6	I–IV, II–V, III–VI	26.27
IX	C-C-C-C-C-C	6	I–IV, II–V, III–VI	1.09
X	CC-C.[PO]C	4	I–IV, II–III	2.33
XI	C-C-CC-CC-C-C	8	I–IV, II–VI, III–VII, V–VIII	2.30
XIV	C-C-C-C	4	I–III, II–IV	3.71

**Table 4:** Performance metrics of the retrained UniDL4BioPep model for conotoxin activity prediction.

Metric	Value
Accuracy (ACC)	0.959
Balanced accuracy (BACC)	0.955
Sensitivity (Sn)	0.969
Specificity (Sp)	0.942
Matthews correlation coefficient (MCC)	0.911
Area under the curve (AUC)	0.991

## Data Availability

The data presented in this study were obtained from publicly available databases, ConoServer (http://www.conoserver.org) (accessed on 1 June 2024) and UniProt (https://www.uniprot.org) (accessed on 1 June 2024). Further details regarding data curation and analysis can be obtained from the corresponding author upon reasonable request.
